# Generalized Mechanism of Field Emission from Nanostructured Semiconductor Film Cathodes

**DOI:** 10.1038/srep43625

**Published:** 2017-03-08

**Authors:** Ru-Zhi Wang, Wei Zhao, Hui Yan

**Affiliations:** 1 College of Materials Science and Engineering, Beijing University of Technology, Beijing 100124, China; 2 State Power Investment Corporation Research Institute, Beijing 102209, China

## Abstract

Considering the effect of both the buffer layer and substrate, a series of ultrathin multilayered structure cathodes (UTMC) is constructed to simulate the field emission (FE) process of nanostructured semiconductor film cathodes (NSFCs). We find a generalized FE mechanism of the NSFCs, in which there are three distinct FE modes with the change of the applied field. Our results clearly show significant differences of FE between conventional emitters and nanofilm emitters, which the non-Fowler-Nordheim characteristics and the resonant FE will be inevitable for NSFCs. Moreover, the controllable FE can be realized by fine-tuning the quantum structure of NSFCs. The generalized mechanism of NSFCs presented here may be particularly useful for design high-speed and high-frequency vacuum nano-electronic devices.

Field emission (FE) have attracted considerable attentions due to their prominent electronic properties, which demonstrate potential applications in flat panel displays and other vacuum microelectronic devices^1^,^2^,^3^,^4^,^5^. Nowadays, the rapid progress of nanotechnology allows the fabrication of nanoscale materials. This development provides many new choices for FE cathodes. The ultrathin film cathodes are developed for high-performance electron field emitters^6^,^7^,^8^, and an ultralow-threshold FE has been observed from nanostructured GaN films in our previous work^9^. The early Fowler-Nordheim (FN) theory^10^ of FE was long successful for some conventional FE materials. However, the FN theory would neglect the effect of the energy band structure from nanoscale. Therefore, the FN theory may be no longer efffective in nanostructured semiconductor film cathodes (NSFCs). For the ultrathin wideband-gap semiconductors (UTSCs) film cathodes^6^, three general *I*-*V* characteristics, including FE-type, T–F-type and explosive emission, have been clearly experimentally observed^6^,^11^,^12^, which may indicate new FE mechanism differing from the conventional FN theory in USFCs.

As a novel FE cathodes, their inner quantum energy-levels induced resonant tunneling dramatically depress the surface effective barrier and Significant improve their FE performance^13^, leading to the possibility of low cost FE nano-device only by the conventional two-dimensional deposition technology. Therefore, NSFCs are extremely promising and have attracted a lot of research interests^14^,^15^,^16^,^17^. Moreover, the resonant tunneling FE induced negative differential conductance (NDC) were found experimental^18^,^19^,^20^ in NSFCs. Unfortunately, such phenomenon cannot be well understood by the conventional FN theory. Recently, we demonstrate the first experimental prototype of high performance FE from nanostructured AlGaN/GaN quantum-well (QW)^13^, making the NSFCs suitable for FE-based applications. However, these interesting experimental results are difficult to explain by conventional FE theory.

In this work, taking into account the buffer layer and substrate of actual FE device, we develop a generalized method of NSFCs by considering the effect of the band structure of quantum-barrier/well, this method is useful for understanding the FE mechanism of NSFCs. We found three electron emission universal modes with different field, which may be originated from the effect of quantum structure in NSFCs. The generalized mechanism presented here are particularly help to design high-performance vacuum nanoelectronic devices by modulating the quantum structure in NSFCs.

## Theoretical Models

III-N materials usually easily crystallize by the wurtzite structure^21^. One of the particularities of this structure for group-III nitrides is always spontaneous electric dipoles along the *c*-axis of the lattice. Along with this spontaneous polarization *P*_SP_ , additional dipoles are created when the material undergoes strain, generating the so-called piezoelectric polarization *P*_PZ_. The total polarization *P*_tot_ = *P*_SP_ + *P*_PZ_ is mutative for different nitrides, thus giving rise to the accumulation of interfacial polarization in nitride-based heterostructures. The built-in polarization causes a strong deformation of the quantum wells accompanied by a strong electrostatic field^22^. As group-III nitrides, Al_*x*_Ga_1−*x*_N (AlGaN) would allow us to control both spontaneous and piezoelectric polarizations by choosing different aluminum compositions. Moreover, by appropriately controlling the aluminum compositions in AlGaN, the potential well depth can be modulated at wide range. Under this circumstance, the quantum structure band shape can be effectively controlled. Therefore, in order to realize various quantum structure of NSFCs, AlGaN are select as nanostructured semiconductor film layer in the present theoretical model.

Since the AlGaN is strained to the buffer layer and band structure can be affected by the substrate, to simulate the band structure of the real FE cathode, both the buffer layer and substrate should be taken into account. On the basis of the aforementioned analysis, we assume that the quantum well cathodes are grown on an n-type (1 × 10^18^ cm^−3^) Si substrate that is 1.0 μm in thickness. On top of this Si substrate are a 200 nm-thick n-type (1 × 10^18^ cm^−3^) GaN (000-1) buffer layer, followed by a 4 nm-thick AlGaN (000-1) potential barrier layer. Finally, a 2 nm-thick GaN (000-1) is grown as well layer.

Generally, the FE current can be given by^23^


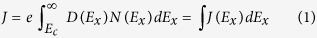


where *E*_*x*_ is the normal energy, *D*(*E*_*x*_) is the transmission coefficient, 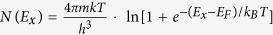 is the supply function, *k*_*B*_ is Boltzmann’s constant, *T* is temperature, *h* is Plank’s constant, and *E*_*F*_ is the Fermi energy. *J*(*E*_*x*_) is the expression of normal-energy distribution written as


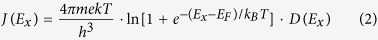


Equation (2) is made up of the transmission coefficient *D*(*E*_*x*_) and the supply function *N*(*E*_*x*_). Transmission coefficient *D*(*E*_*x*_) can be calculated by the transfer matrix (TM) method in our previous work^24^. In the TM method for computing *D*(*E*_*x*_), potential barrier shape is a key parameter affecting the transmission coefficient dramatically. Herein, a more complicated and realistic image potential involving the image potential shifting was introduced. The parameters are the same as in our previous work^24^.

## Results and Discussions

**Figure 1:**
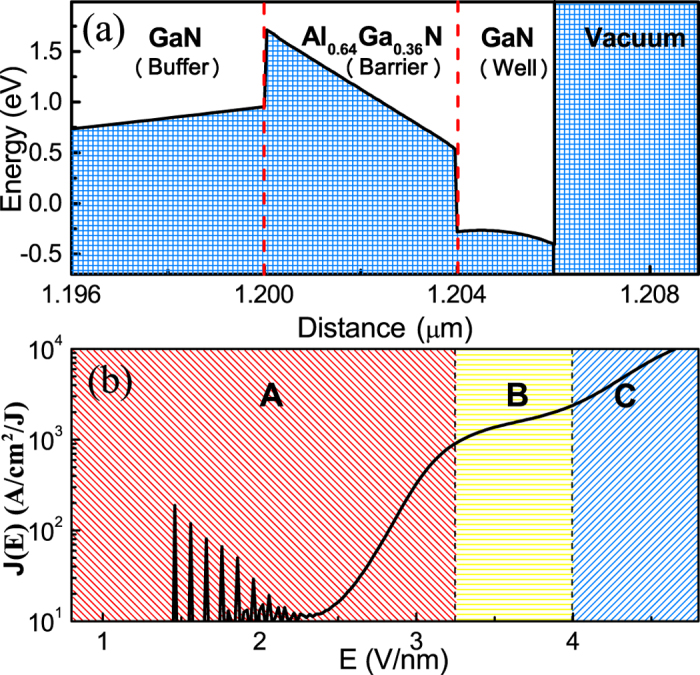
(**a**) Energy band diagram of multilayered structure cathodes (Al_0.64_Ga_0.36_N/GaN) without bias, and the corresponding FE characteristics: (**b**) FE current density as a function of the applied electric field (*J*-*E*).

Due to the electron confinement in the QW, the quantum energy-levels induced resonant tunneling dramatically depress the surface effective barrier and significantly improve FE properties^15^. In order to effectively confine the electron in the QW, the difference in the energy gap ΔE_g_ between the QW and the quantum barrier (QB) layer are fixed at 1.6 eV. As a result, Al_0.64_Ga_0.36_N ternary nitride is used as QB layer. The energy band diagrams of the Al_0.64_Ga_0.36_N/GaN (4 nm/2 nm) quantum-well are shown in Fig. 1(a). It can be found that the conduction bands are strongly deformed when the built-in polarization is considered. It is noted that the energy barrier height created by Al_0.64_Ga_0.36_N is substantially increased by the high density of positive polarization charges *C*_*p*_ (3.5 × 10^13^ cm^−2^) at the interface between the GaN buffer layer and the Al_0.64_Ga_0.36_N QB layer. Under this condition, the electrons are attracted by a Coulomb force and accumulate at this interface (AlGaN/GaN (well/barrier)), which may leads to a strong band bending.

Figure 1(b) shows the characteristic of the FE current density variation versus the applied field for the Al_0.64_Ga_0.36_N/GaN quantum-barrier/well. The semilog plot shows three characteristics regions for different field. In the region A, which is at the beginning of the electron emission, there are many FE oscillation peaks, which is similar to a resonant FE behavior. In the region B, it shows an increase in the current which is distinct different from that of the region A. In the region C, a very steep increase of the current has been found. Such three type of FE are similar to the *I*-*V* characteristics obtained by UTSCs^6^. However, the relative FE mechanism is not clear, especially for the different structure of FE cathode with different field, a generalized FE mechanism of USFCs is further needed to develop.

**Figure 2:**
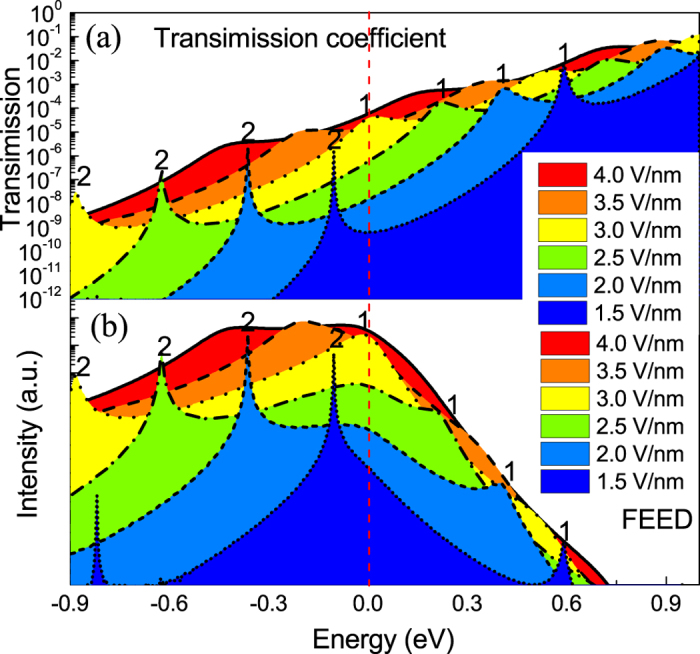
Illustration of the (**a**) electron transmission coefficient and (**b**) FEED of the multilayered structure cathodes (Al_0.64_Ga_0.36_N/GaN) under different external electric field.

Here, field emission energy distribution (FEED) was firstly used to explore the novel FE mechanisms. Figure 2 shows the calculated variation in the electron transmission and FEED for Al_0.64_Ga_0.36_N/GaN. It is obvious that there are two distinct peaks when electrons tunnel through the dual-barrier potential well, indicating that two quantum energy-levels localize in the USFCs. The electrons are emitted by a FE mechanism from the quantized subbands ins the quantum-well. With increasing of the fields, the entire transmission increases by orders and shift toward low energy sides, which origins from strong band bending. Base on the calculated variation in the *J*-*E* and FEED characteristics, we presented three field electron emission modes.

### Resonant-tunneling-type field emission (RT-FE)

At a field below 2.5 V/nm, electrons origins from 2# quantum energy-level near the Fermi level *E*_*F*_ and resonant tunnel through the vacuum barrier, there are some resonant FE peaks. When the external electric field ranging from 2.5 to 3.0 V/nm, the 1# quantum energy-level, which is far from *E*_*F*_ in the low-field, gradually shift toward *E*_*F*_. Due to the dramatic increase of the electron supply when the energy shift from above *E*_*F*_ to *E*_*F*_, the electrons origins from both 1# and 2# quantum energy levels when the field is at 2.5–3.0 V/nm. Thus FE current increases remarkably, corresponding to the region A in Fig. 1(b).

### Saturated Fowler-Nordheim field emission (S-FN)

As the applied field is increased above 3.0 V/nm, 1# quantum energy-level shift from *E*_*F*_ to below the *E*_*F*_. Since the electron transmission of 1# quantum energy-level decrease with the increase of the applied field as shown in Fig. 2, considering the effect of electron transmission with electron supply, FEED peak of 1# quantum energy-level are increased tardily rather than that of the sharp increase at the lower field (from 2.5 to 3.0 V/nm), leading to the FE current increase slowly as shown in region B in the corresponding *J*-*E* curve.

### Mixed Thermal-FN field emission (Mixed T-FN)

When further increased external electric field beyond 4.0 V/nm, due to the vacuum-level fall to the *E*_*F*_, the FE electrons can directly tunnel through the single AlGaN barrier. As a result, the electron transmissions are dramatically promoted. Moreover, since the electrons cannot be effectively confined in the QW, and the quantized subbands disappeared, as well as the FEED peaks in the Fig 2. Therefore, FE modes changing from S-FN to T-FN, corresponding to the region C in Fig. 1(b). If we regard AlGaN QB as vacuum barrier, such mode is similar to mixed thermal and FN field electron emission.

## Structure Effects For The Multilayered Cathodes

In order to confirm that such three field electron emission modes are universal for all USFCs, the effects of quantum-barrier/well width, quantum structure band shape, and quantum-well depth on the USFCs were also investigated, due to the limit paper, some important and necessary results were presented in the following part. For more results, it can also be found in the support information (Figs S1–S15).

### Quantum-barrier/well width effects

**Figure 3:**
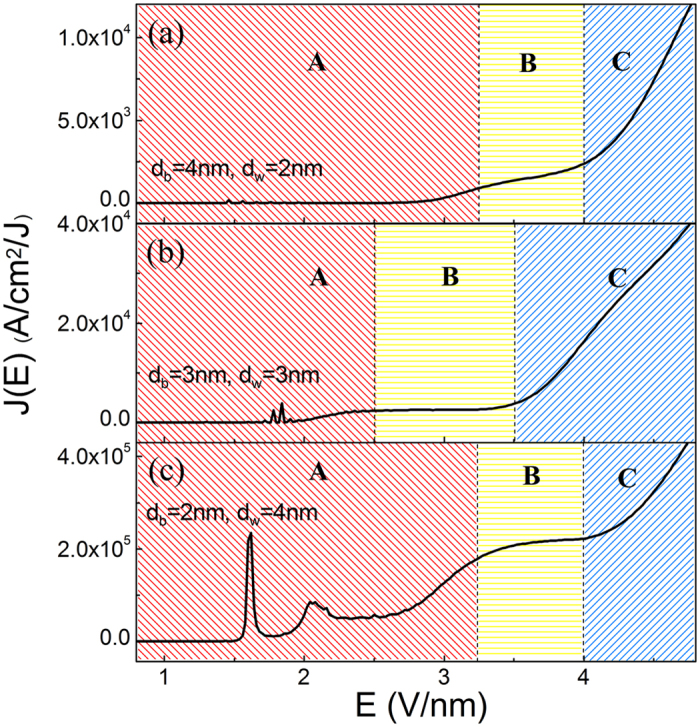
*J-E* curves of multilayered structure cathodes (Al_0.64_Ga_0.36_N/GaN) with the change in layer thickness proportion, (**a**) 4 nm/2 nm, (**b**) 3 nm/3 nm, and (**c**) 2 nm/4 nm.

**Figure 4:**
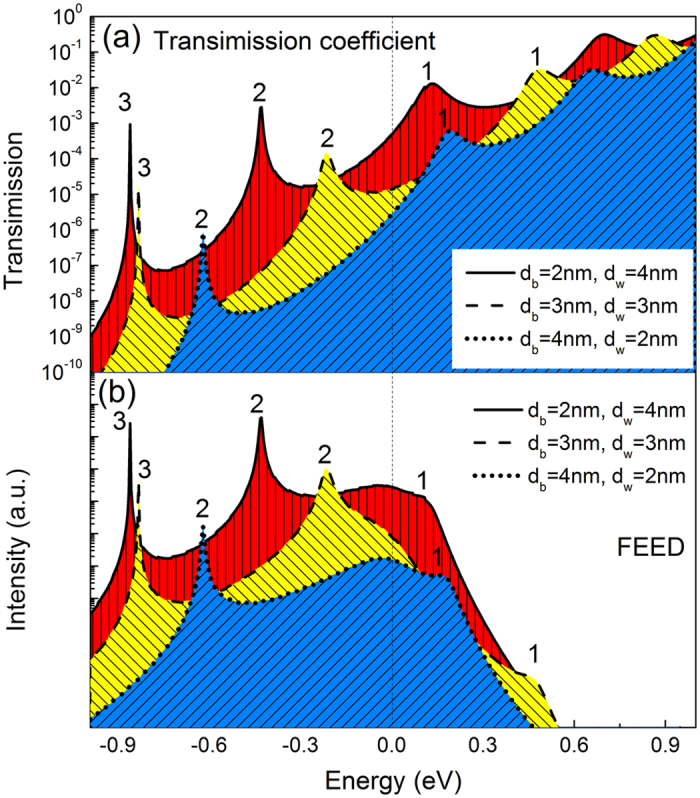
Evolution of the (**a**) Electron transmission coefficient and (**b**) FEED of multilayered structure cathodes with the change in layer thickness proportion at the field of 2.0 V/nm.

Figure 3 shows the characteristic of the emission current density versus the applied field from the Al_0.64_Ga_0.36_N/GaN quantum-barrier/well with different layer thickness proportion, where the total thickness of the AlGaN/GaN films are kept at 6 nm. It is clear that, three general FE modes have been observed with the change of the field. Calculated results shown that, with the change of the quantum structure, the *I*-*V* characteristic is distinctly changed. Moreover, Fig. 4 reveals the electron transmission coefficient and FEED of 6 nm Al_0.64_Ga_0.36_N/GaN quantum-barrier/well with the change in layer thickness proportion. It is visually obvious that the electron transmission coefficient is dramatically affected by the width of the QW. In addition, not only the magnitude of the transmission coefficients but also the positions of FEED peaks can be changed tremendously when the individual layer thicknesses of the FE structure are modulated. It is found that the difference in the neighbor quantum energy-levels ΔE_s_ increase remarkably as the width of QW decreases.

By comparing the FEED of these three FE configurations, it is easily found field electron emission changing from single-energy-level to multi-energy-levels. It can be approved clearly in Fig. 4 that there are larger ΔE_s_ in the QW for having the lower QW width, and the larger ΔE_s_ lead to the less quantum energy-level. In the case of the structure of 4 nm/2 nm, near the Fermi level (at zero position), there has only one quantum energy-level, which plays a major contribution to the FE current. On the other hand, the larger QW width leads to the decrease of ΔE_s_ in the QW, and the decreasing ΔE_s_ lead to the more quantum energy-level near the Fermi level, which may supply more FE electron. The above phenomenon was also supported by the experimental observations from Johnson^19^ and Kildemo^25^ *et al*. In addition, compared with single-energy-level electron emission, multi-energy-levels electron emission is sluggish response to external electric field, thus few resonant FE current peaks can be found from the FE *J*-*E* curves. Such distinct resonant FE current peaks act as negative differential conductance, as can be seen from Fig. 3(c), and similar experimental factss have been found^13^,^18^,^19^.

### Band structure shape effects

In order to investigate the band structure effect, the band shape or the well depth should be modulated individually. Recently, an approach to control the electrostatic fields by using quaternary Al_x_In_y_Ga_1−x−y_N (AlInGaN) layers could be an attractive alternative for *c*-plane GaN-based heterostructures since the introduction of quaternary AlInGaN layers would allow us to control both spontaneous and piezoelectric polarizations by choosing different aluminum and indium compositions^8^,^26^. Therefore, by appropriately controlling the aluminum and indium compositions in AlInGaN, the built-in charge density at the interface between GaN and AlInGaN barrier can be adjusted. Under this circumstance, the quantum structure band shape can be effectively modulated.

**Figure 5:**
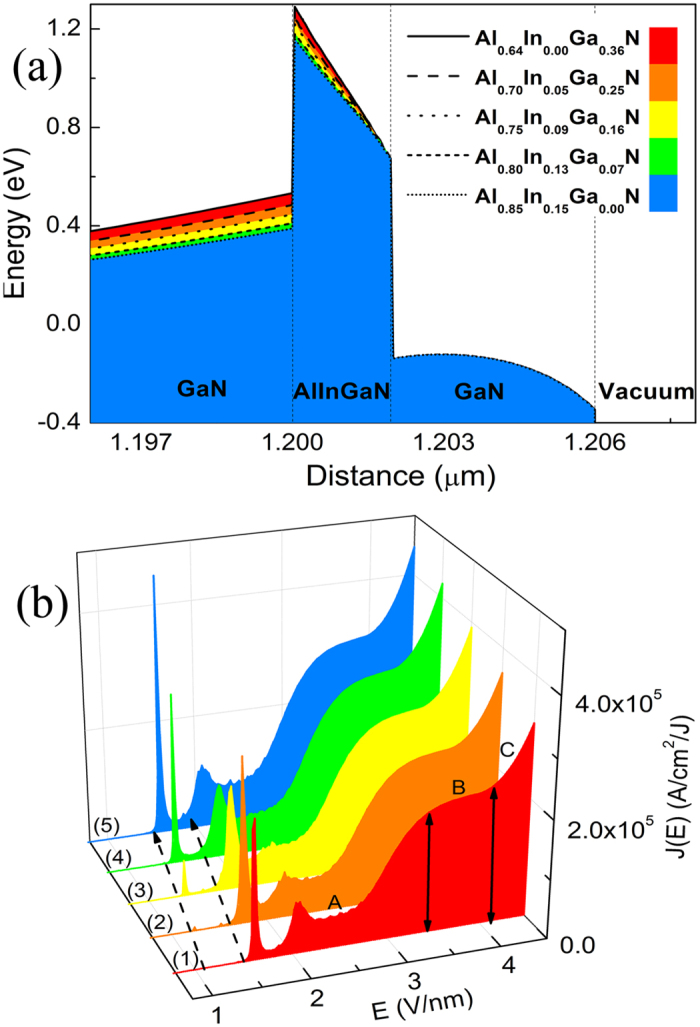
Evolution of the (**a**) energy band diagram of multilayered structure cathodes under different composition without bias, the Δ*E*_*g*_ are fix at 1.6 eV, and the corresponding (**b**) *J-E* curves of 2 nm/4 nm multilayered structure cathodes under different composition of the barrier layer, nos. 1 to 5 are Al_0.64_In_0.00_Ga_0.36_N, Al_0.70_In_0.05_Ga_0.25_N, Al_0.75_In_0.09_Ga_0.16_N, Al_0.80_In_0.13_Ga_0.07_N, and Al_0.85_In_0.15_Ga_0.00_N, respectively.

Base on the structure of 2 nm/4 nm quantum-barrier/well that mentioned above, by fine-tuning different aluminum and indium compositions of the quaternary AlInGaN layers, the band structure shape were effectively controlled by the way of built-in charge density modulating at the interface. And at the same time the QW depth was not changed. Seeing energy band of the quantum structure for different QB quaternary composition in Fig. 5(a), it is obvious that the AlInGaN barrier is evidently lowered by increasing Al composition. So in Fig. 5(b), we calculated the FE characteristic with the change of the applied field. It can be seen clearly that the FE properties is greatly improved with increasing applied field. The results also indicate that the three general FE modes were not influenced by the change of band structure shape. However, it is obvious that the resonant FE current peaks can shift toward low-field when the shape of quantum structure band is modulated.

### Quantum-well depth effects

**Figure 6:**
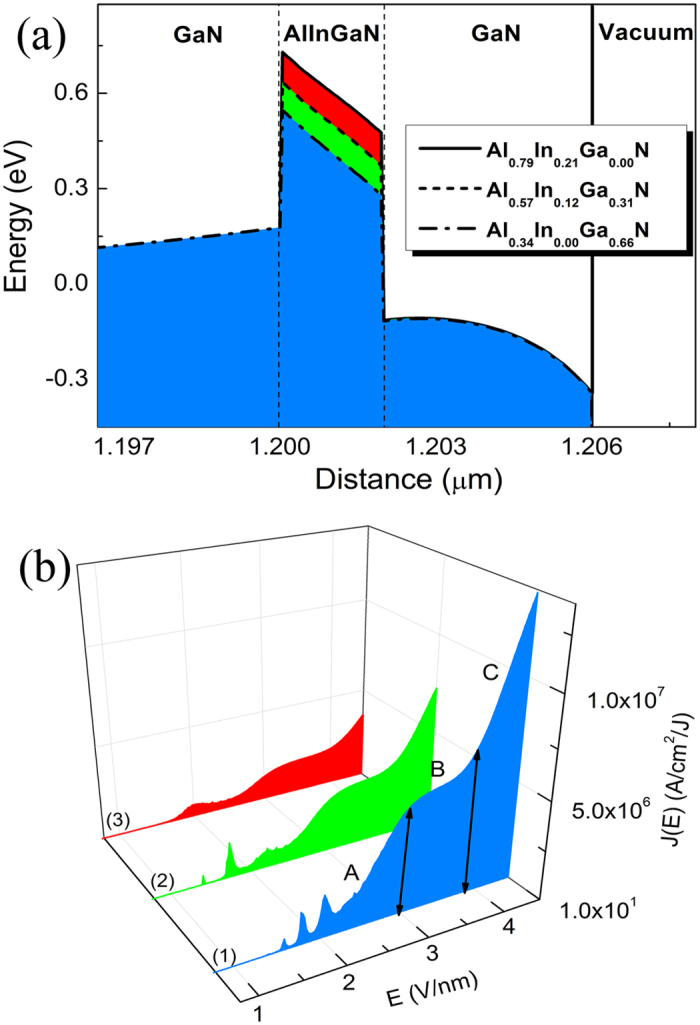
Evolution of the (**a**) energy band diagram of multilayered structure cathodes under different composition without bias, the *C*_*p*_ are fix at 1.6 × 10^13^ cm^−2^, and the corresponding (**b**) *J-E* curves of multilayered structure cathodes under different composition, nos. 1 to 3 are Al_0.34_In_0.00_Ga_0.66_N, Al_0.57_In_0.12_Ga_0.31_N, and Al_0.79_In_0.21_Ga_0.00_N, respectively.

In order to better understand the effect of quantum-well depth, the AlInGaN layer was used to control the potential well depth by the method of compositions modulation and keeping the built-in charge density in the interface unchanged (*C*_*p*_ = 1.6 × 10^13^ cm^−2^). Base on the calculated variation in the *J*-*E* [Fig. 6(b)] and their FEED characteristics (not shown here), it is indicates that the three general FE modes are not influenced by the change of quantum-well depth. And the FE *J*-*E* curves show that, although the FE current density were reduced with increasing the depth of the QW, the number of resonant FE current peaks were remarkably reduced and the intensity of resonant FE current peaks were dramatically promoted, and single resonant FE current peak is distinctly observed.

As it is well known, the operational speed of solid state microelectronic devices is hampered by the saturation velocity of electrons^27^. Combined with the band shape and QW depth modulation, the prominent NDC characteristic of NSFCs in the low-field are extremely promising for operation high-speed electronics, making the generalized mechanism presented here particularly useful for design high-speed and high-frequency vacuum microelectronic devices. In particular, the electron velocity in vacuum can approach the speed of light, which are far faster nearly three orders of magnitude than that of solid state electronic devices, making the FE-based devices suitable for high-speed and high-frequency applications.

## Conclusions

In summary, considering the effect of both the buffer layer and substrate, we present a generalized FE mechanism for the NSFCs. This generalized FE mechanism can be divided into three electron emission modes, including resonant-tunneling-type field emission in the low-field, saturated Fowler-Nordheim field emission, and Mixed Thermal-FN field emission in the high-field. Moreover, by modulating the quantum-barrier/well width, band structure shape, and quantum-well depth of NSFCs, three general FE modes still obviously exist. In particular, the NSFCs is a special structure of ultrathin multilayered structure cathodes. Therefore, this mechanism can be considered as describing the novel physical properties of FE of NSFCs. It is also found that the electron emission characteristics of these NSFCs can be modulated by engineering the quantum structure. In addition, by fine-tuning the shape and depth of the quantum structure at a proper QB/QW width, single resonant FE current peak can be achieved at low-field, making it suitable for high-speed and high-frequency vacuum microelectronic devices.

## Additional Information

**How to cite this article**: Wang, R.-Z. *et al*. Generalized Mechanism of Field Emission from Nanostructured Semiconductor Film Cathodes. *Sci. Rep.* **7**, 43625; doi: 10.1038/srep43625 (2017).

**Publisher's note:** Springer Nature remains neutral with regard to jurisdictional claims in published maps and institutional affiliations.

## Supplementary Material

Supplementary Information
