# Supplementary material for: Generalized Mechanism of Field Emission from Nanostructured Semiconductor Film Cathodes
Source: Sci Rep. 2017 Mar 8;7:43625. doi: 10.1038/srep43625 (PMC5343652; doi:10.1038/srep43625)
Supplement: Supplementary Information [file srep43625-s1.pdf]

Supplementary Information

Generalized Mechanism of Field Emission from Nanostructured Semiconductor Film Cathodes

Ru-Zhi Wang[1*], Wei Zhao[2], and Hui Yan[1]

1) College of Materials Science and Engineering，Beijing University of Technology, Beijing 100124, China

2) State Power Investment Corporation Research Institute, Beijing, 102209, China

Supplementary Information Guide

This file includes

Supplementary Figures and Legends S1-S15

* To whom correspondence should be addressed: wrz@bjut.edu.cn.

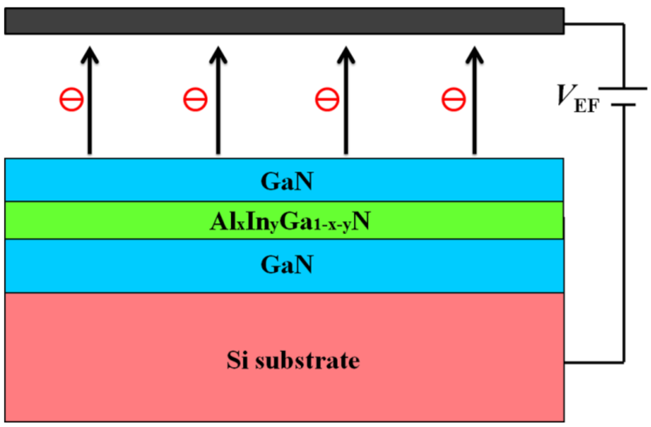

Fig. S1 Schematic representation of Nanostructured Semiconductor Film Cathodes (NSFCs)

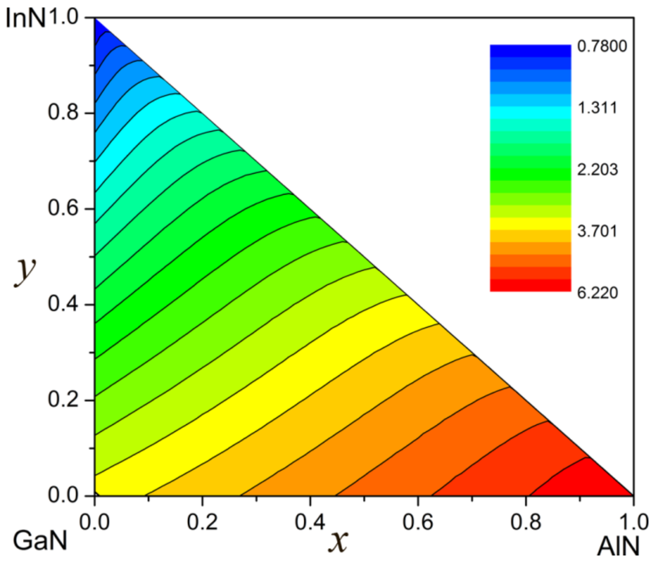

Fig. S2 Calculated energy gap for $Al_xIn_yGa_{1-x-y}N$ as the barrier composition varies

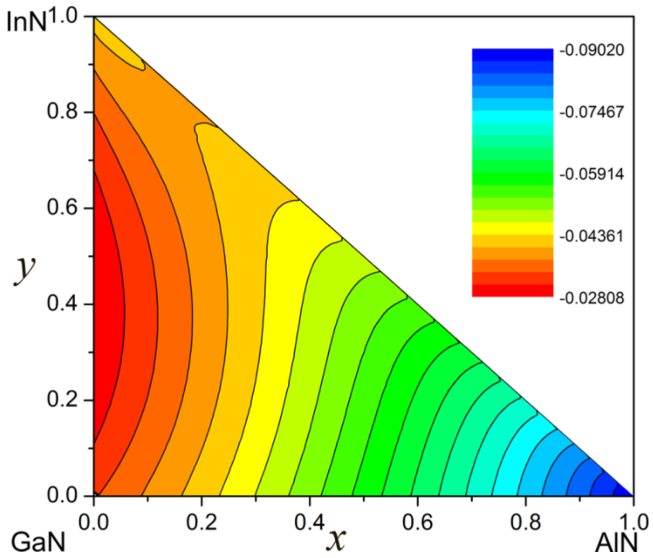

Fig. S3 Calculated spontaneous polarization for $Al_xIn_yGa_{1-x-y}N$ as the barrier composition varies

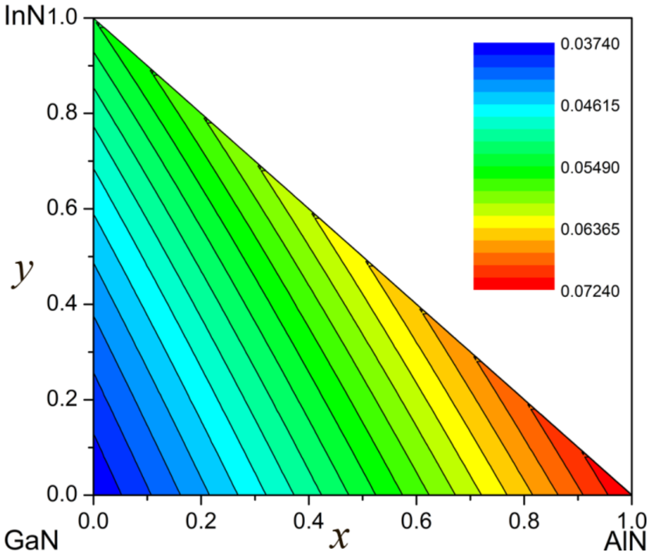

Fig. S4 Calculated piezoelectric polarization for $Al_xIn_yGa_{1-x-y}N$ as the barrier composition varies

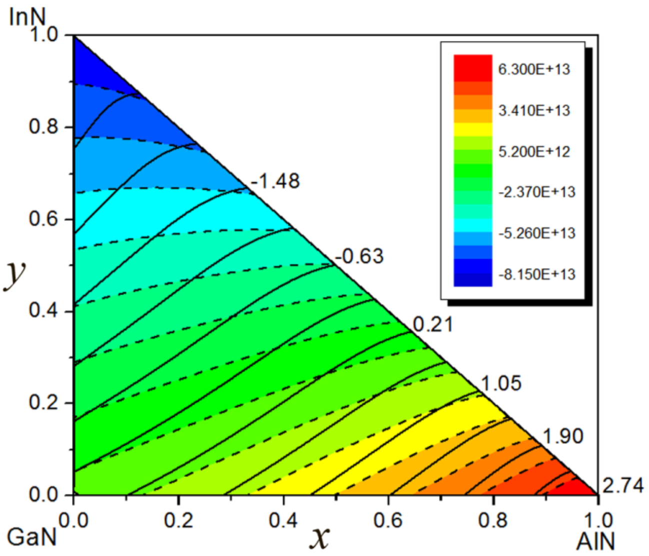

Fig. S5 Calculated polarization charge C_p and band gap difference ΔE_g for $Al_xIn_yGa_{1-x-y}N$/GaN interface as the barrier composition varies. The dashed lines denote the C_p and the thick solid lines indicate the ΔE_g between well and barrier

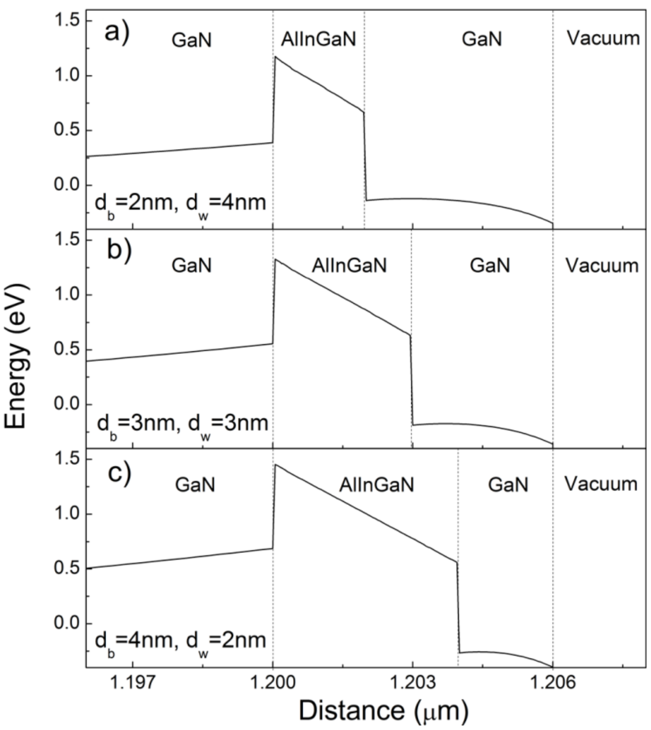

Fig. S5 Evolution of the energy band diagram of $Al_{0.85}In_{0.15}Ga_{0.00}N$/GaN under different barrier/well thickness without bias, a) 2nm/4nm,b) 3nm/3nm, and c) 4nm/2nm

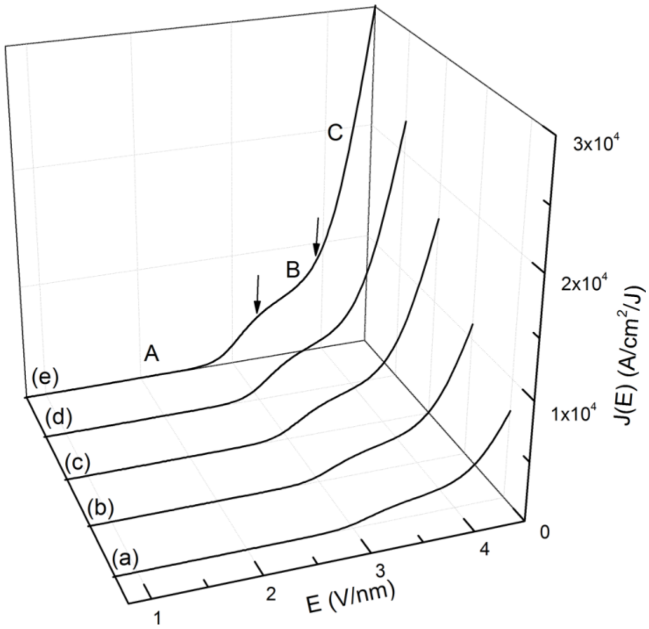

Fig.S6 Evolution of the *J-E* curves of 4nm/2nm barrier/well structure films under different composition, a) $Al_{0.64}In_{0.00}Ga_{0.36}N$, b) $Al_{0.70}In_{0.05}Ga_{0.25}N$, c) $Al_{0.75}In_{0.09}Ga_{0.16}N$, d) $Al_{0.80}In_{0.13}Ga_{0.07}N$, and e) $Al_{0.85}In_{0.15}Ga_{0.00}N$

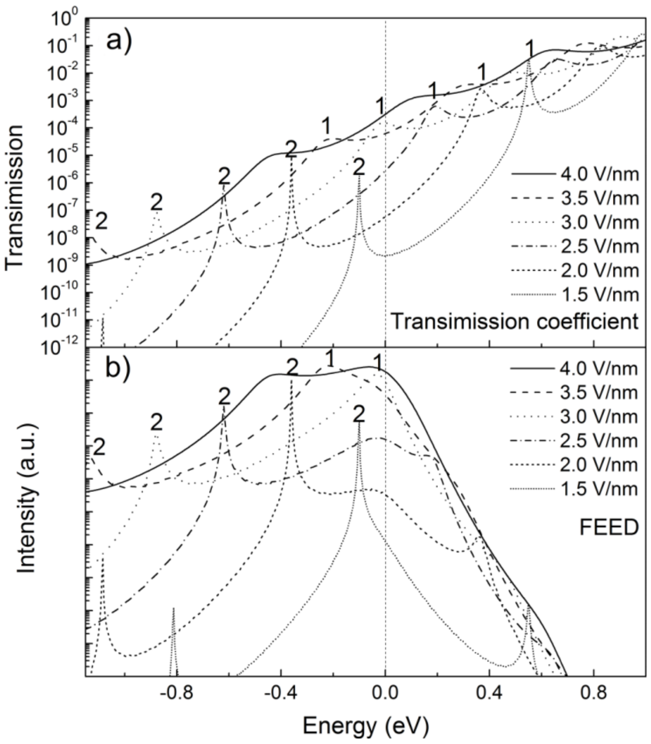

Fig S7 Evolution of the a) electron transmission coefficient and b) FEED of 4nm/2nm barrier/well structure films under different applied field

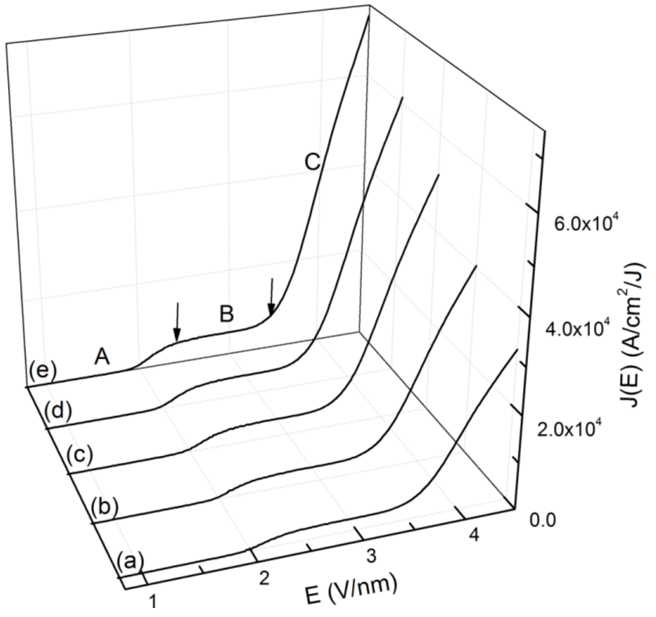

Fig. S8 Evolution of the *J-E* curves of 3nm/3nm barrier/well structure films under different composition, a) $Al_{0.64}In_{0.00}Ga_{0.36}N$, b) $Al_{0.70}In_{0.05}Ga_{0.25}N$, c) $Al_{0.75}In_{0.09}Ga_{0.16}N$, d) $Al_{0.80}In_{0.13}Ga_{0.07}N$, and e) $Al_{0.85}In_{0.15}Ga_{0.00}N$

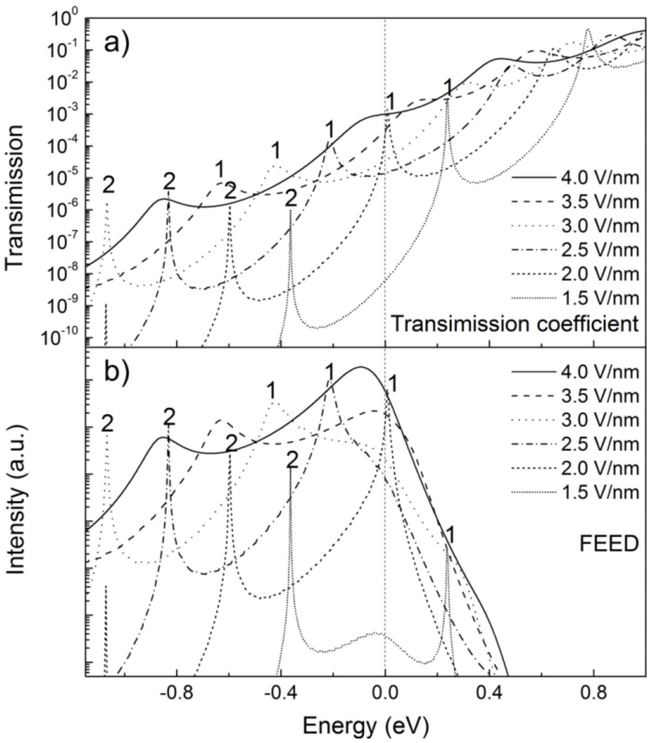

Fig. S9 Evolution of the a) electron transmission coefficient and b) FEED of 3nm/3nm barrier/well structure films under different applied field

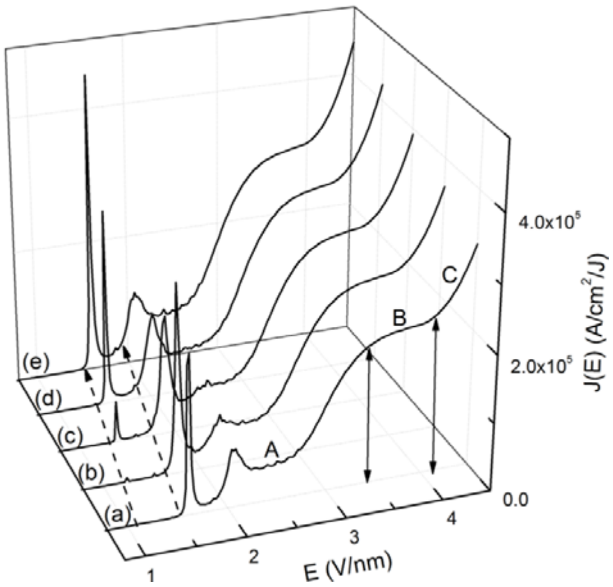

Fig. S10 Evolution of the *J-E* curves of 2nm/4nm barrier/well structure films under different composition, a) $Al_{0.64}In_{0.00}Ga_{0.36}N$, b) $Al_{0.70}In_{0.05}Ga_{0.25}N$, c) $Al_{0.75}In_{0.09}Ga_{0.16}N$, d) $Al_{0.80}In_{0.13}Ga_{0.07}N$, and e) $Al_{0.85}In_{0.15}Ga_{0.00}N$

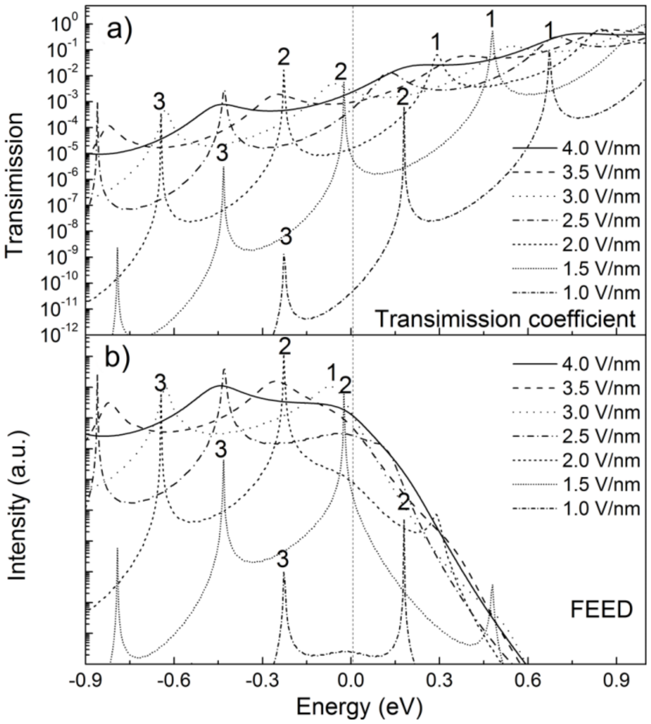

Fig. S11 Evolution of the a) electron transmission coefficient and b) FEED of 2nm/4nm barrier/well structure films under different applied field

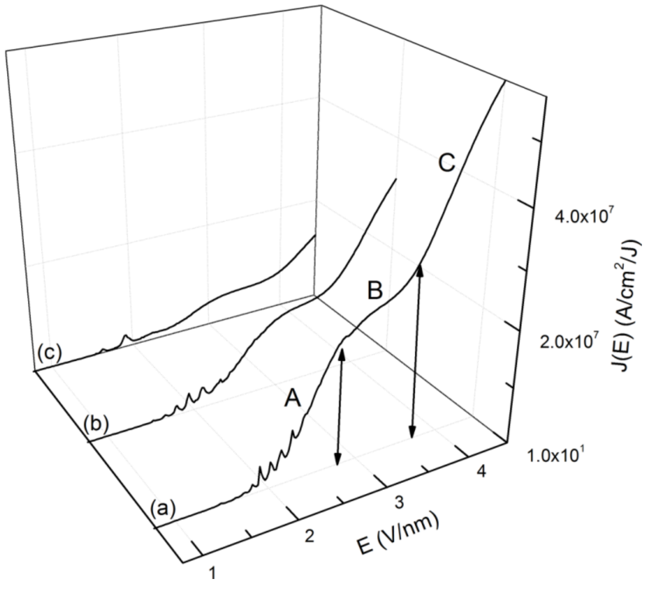

Fig. S12 Evolution of the *J*-*E* curves of quantum structure films under different composition for the C_p of 0.8×10^{13} cm^{-2}, a) $Al_{0.18}In_{0.00}Ga_{0.82}N$, b) $Al_{0.45}In_{0.13}Ga_{0.42}N$, and c) $Al_{0.75}In_{0.25}Ga_{0.00}N$

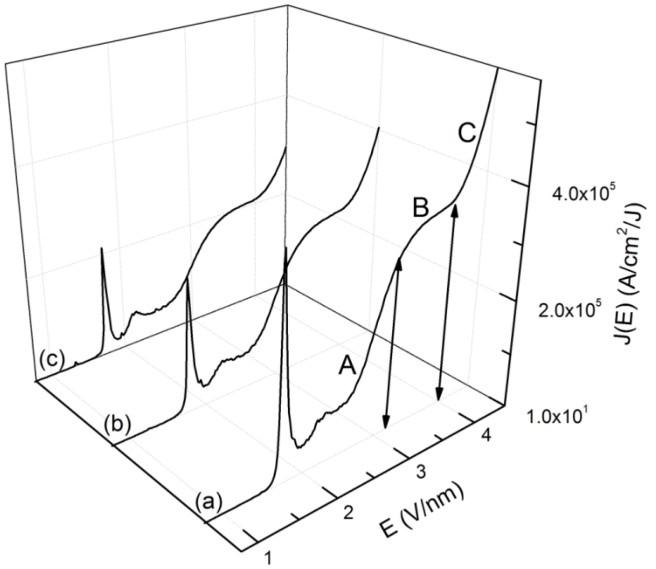

Fig. S13 Evolution of the *J-E* curves of quantum structure films under different composition for the C_p of 3.2×10^{13} cm^{-2}, a) $Al_{0.59}In_{0.00}Ga_{0.41}N$, b) $Al_{0.72}In_{0.07}Ga_{0.21}N$, and c) $Al_{0.86}In_{0.14}Ga_{0.00}N$

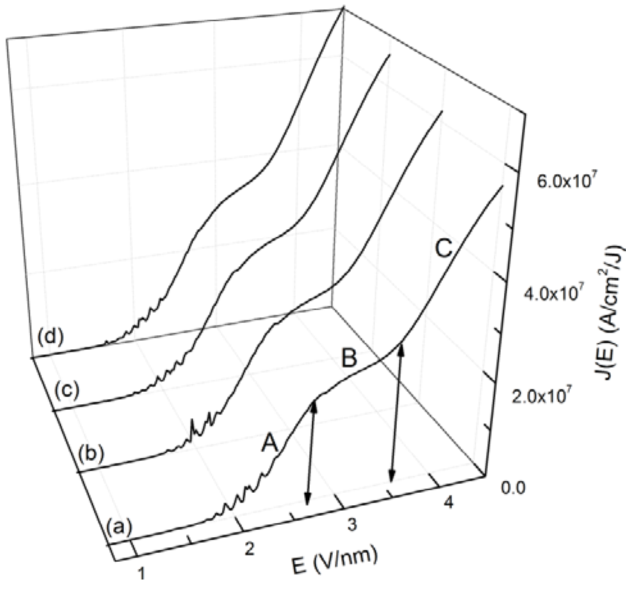

Fig. S14 Evolution of the *J-E* curves of quantum structure films under different composition for the ΔE_g of 0.4 eV, a) $Al_{0.19}In_{0.00}Ga_{0.81}N$, b) $Al_{0.33}In_{0.10}Ga_{0.57}N$, c) $Al_{0.50}In_{0.23}Ga_{0.27}N$, and d) $Al_{0.67}In_{0.33}Ga_{0.00}N$

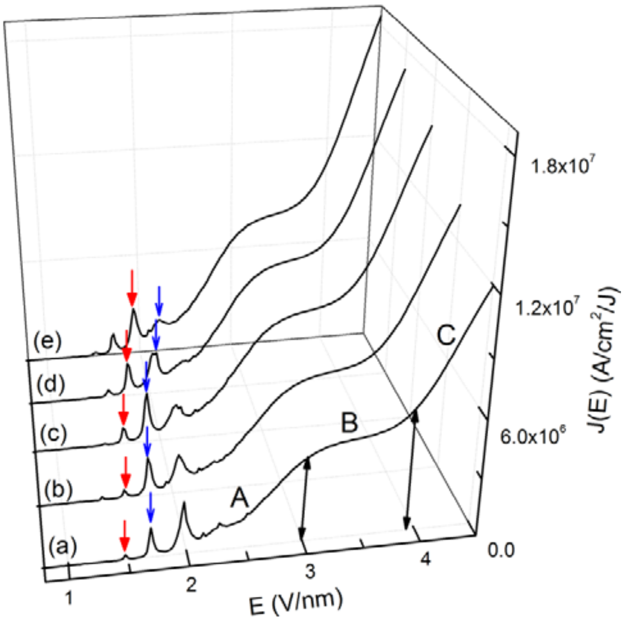

Fig. S15 Evolution of the *J*-*E* curves of quantum structure films under different composition for the ΔE_g of 0.8 eV, a) $Al_{0.35}In_{0.00}Ga_{0.65}N$, b) $Al_{0.44}In_{0.07}Ga_{0.49}N$, c) $Al_{0.54}In_{0.15}Ga_{0.31}N$, d) $Al_{0.64}In_{0.21}Ga_{0.15}N$, and e) $Al_{0.73}In_{0.27}Ga_{0.00}N$
